# Brain lymphatic drainage pathways, deep cervical lymphatic surgery, and current insights: A systematic review

**DOI:** 10.1016/j.tjpad.2025.100335

**Published:** 2025-08-27

**Authors:** Theodore Lahmar, Francois Thuau, Gaelle Pinard, Claire Boutoleau-Bretonniere, Pierre Perrot, Ugo Lancien

**Affiliations:** a Department of Plastic and Reconstructive Surgery, University Hospital of Nantes, 1 place Alexis Ricordeau 44000 Nantes, France; b RMeS: Regenerative Medicine and Skeleton research unit - Inserm, Nantes University 44000 Nantes, France; c Department of Neurology, University Hospital of Nantes, CIC1314 INSERM 44000 Nantes, France

**Keywords:** Glymphatic, Meningeal lymphatics, Brain lymphatics, Lymphatic surgery, Lymphatico-venous anastomosis, Supermicrosurgery, Alzheimer disease, Parkinson disease, Dementia surgery

## Abstract

The discovery of the glymphatic system and the later rediscovery of the meningeal lymphatic network have significantly changed our understanding of central nervous system (CNS) waste clearance. Aging is linked to a gradual decline in these clearance pathways, resulting in waste buildup. As a result, therapeutic strategies targeting cerebral lymphatic function have garnered growing interest, with lymphatic surgery emerging as a promising option.

We conducted a review until July 2025, providing an overview of the potential of lymphatic surgical techniques to enhance CNS metabolic waste clearance pathways as a therapeutic approach for brain lymphatic system disorders.

Currently available data are limited, nine publications addressing this approach. These studies explore an innovative technique involving lymphatico-venous anastomoses (LVA) targeting deep cervical lymphatic vessels to promote clearance for the treatment of Alzheimer’s or Parkinson’s diseases.

Cerebral lymphatic drainage is critical for effective brain waste elimination such as amyloid-β, phosphorylated tau, and α-synuclein, which are linked to neurodegenerative diseases. Viewing these lymphatic dysfunctions as a form of “cerebral lymphedema,” LVA, already used in treating peripheral lymphedema, shows potential as a therapeutic approach. Although clinical evidence is still limited, lymphatic supermicrosurgery presents promising therapeutic possibilities for neurodegenerative diseases and other conditions related to impaired CNS lymphatic outflow.

## 1 Introduction

The description of the glial-lymphatic or glymphatic system by Iliff et al. (2012) transformed our understanding of brain waste clearance mechanisms. This system comprises a dynamic process that involves the circulation of interstitial fluid (ISF) and cerebrospinal fluid (CSF), playing a crucial role in eliminating neurotoxic macromolecules such as amyloid-β and tau protein [1]. Subsequently, Louveau et al and Aspelund et al independently rediscovered a network of meningeal lymphatic vessels that facilitate CSF drainage into deep cervical lymph nodes (dcLNs), reinforcing the essential role of the peripheral lymphatic system in cerebral waste clearance [2,3].

Since these discoveries, extensive research has highlighted the importance of the lymphatic network in clearing cerebral metabolites and maintaining cerebral homeostasis. Impairment of these lymphatic routes has been linked to the accumulation of macromolecules, accelerating the progression of neurodegenerative disorders such as Alzheimer’s disease (AD) and idiopathic Parkinson’s disease (PD) [4,5].

Aging has been recognized as a primary risk factor for the dysfunction of cerebral drainage pathways. Experimental studies have demonstrated that aging significantly declines CSF lymphatic clearance capacity, contributing to the progressive accumulation of toxic macromolecules within the brain parenchyma [4,6,7]. This decline is thought to be a key factor in the pathophysiology of age-related neurodegeneration and cognitive decline.

The increasing interest in the lymphatic system as a therapeutic target has led to the creation of innovative strategies aimed at enhancing or restoring lymphatic drainage within the central nervous system (CNS). Notably, a study by Du et al. demonstrated that applying prostaglandins to the cervical lymphatic vessels of aged mice restores lymphatic flow and improves cerebral waste clearance [8]. These findings highlight the potential of targeted interventions to counteract age-related impairments in brain waste clearance. Concurrently, recent advancements in surgical lymphatic reconstruction, particularly for treating lymphatic congestion disorders like lymphedema, encourage us to consider whether similar methods could be utilized to improve the function of cervical lymphatic pathways that are responsible for CNS drainage.

This study offers a thorough synthesis of existing knowledge on CNS lymphatic drainage, assesses emerging surgical strategies involving lymphatic shunting in the management of Alzheimer’s and Parkinson’s diseases through a systematic review, critically evaluates the available evidence, and addresses key challenges, limitations, and future therapeutic opportunities in the field.

## 2 Methods

### 2.1 Study design

This review adheres to the methodological recommendations specified by the Joanna Briggs Institute, [9] and follows Preferred Reporting Items for Systematic Review and Meta-analyses (PRISMA) guidelines [10]. The review seeks to clarify key concepts, synthesize research findings, and identify gaps to guide future research. A protocol was developed and registered in the PROSPERO database (CRD420251086139) to promote methodological transparency and alignment with recognized standards for systematic reviews.

### 2.2 Search strategy

In July 2025, a search was conducted across PubMed, the Cochrane Library, and supplemented by Google Scholar. The search was restricted to studies published from 2015 onward, following the rediscovery of meningeal lymphatic vessels, which marked a turning point in the field. The search strategy incorporated a wide range of key terms to capture all potentially relevant and were based on the MeSH (Medical Subject Headings) thesaurus. The following terms were used: ((Microsurgery) OR (LVA) OR (LNVA) OR (Lymphatic Shunt) OR (Anastomosis) OR (Lymphaticovenous anastomosis) OR (Lymphovenous anastomosis) OR (lymphatic surgery) OR (Lymphatic bypass) OR (Supermicrosurgery) OR (Deep cervical lymphatic) OR (Meningeal lymphatic) OR (Cervical lymphatic)) AND ((neurodegenerative diseases) OR (Cognitive Dysfunction) OR (Glymphatic) OR (Alzheimer) OR (Parkinson) OR (Brain lymphatic) OR (Cerebral lymphatic) OR (Alzheimer’s disease) OR (Parkinson’s disease) OR (Brain) OR (Central Nervous System)).

To ensure comprehensive coverage of existing literature, the references of retrieved articles were manually screened to identify additional relevant studies.

### 2.3 Eligibility criteria

Studies were included if they investigated lympho-venous bypass procedures for restoring CNS lymphatic drainage pathways, were conducted in humans, and addressed conditions relevant to Alzheimer’s or Parkinson’s disease. All study designs were eligible, including randomized controlled trials, non-randomized controlled trials, case series, case reports, observational studies (retrospective or prospective), and reviews. Studies were excluded if they were unrelated to the review question, involved animal or cadaveric models or focused on other neurological or neurodegenerative disorders unrelated to Alzheimer's or Parkinson's disease, or were reviews without original data. There were no limits on the search; if foreign language articles were identified, every effort was made to obtain English copies or to translate the article.

### 2.4 Study selection process

The selection process included two phases by two independent researchers. Disagreements were resolved through discussion or, if necessary, by a third reviewer. First, duplicate records were removed, and articles were screened based on their titles and abstracts to evaluate relevance. Second, potentially relevant studies underwent a full-text review to verify eligibility.

Most of the selected articles were accessible online; for those with restricted access, corresponding authors were contacted to request full texts.

### 2.5 Data extraction and synthesis

All articles identified through electronic and manual searches were listed with key information using Microsoft Excel (Microsoft Corp., Redmond, WA, USA). Data from the included studies were extracted and synthesized in a narrative format including study characteristics, patient demographics, intervention details, evaluated outcomes and reported limitations.

### 2.6 Quality assessment

Two independent reviewers assessed the methodological quality and risk of bias of the included clinical studies. The evaluation was conducted using the MINORS tool (Methodological Index for Non-Randomized Studies), and Joanna Briggs Institute (JBI) Critical Appraisal Checklist for Case Reports. Any disagreements were resolved through discussion or, if necessary, consultation with a third reviewer.

## 3 Results

### 3.1 Study selection

A total of 4712 references were retrieved using the predefined search terms across two structured databases: PubMed (*n* = 4099), Cochrane Library (*n* = 201), and an additional search using Google Scholar (*n* = 412). An additional, five articles were identified through manual screening of reference lists from relevant studies.

After removing 52 duplicates, 4665 titles and abstracts were screened. At this stage, 4642 articles were excluded for not meeting the inclusion criteria, and 23 full-text articles were retrieved for in-depth evaluation. Following full-text analysis, 14 articles were excluded for the following reasons: unavailable full text (*n* = 3), animal-based studies (*n* = 2), and lack of original data (*n* = 9).

**Fig. 1:**
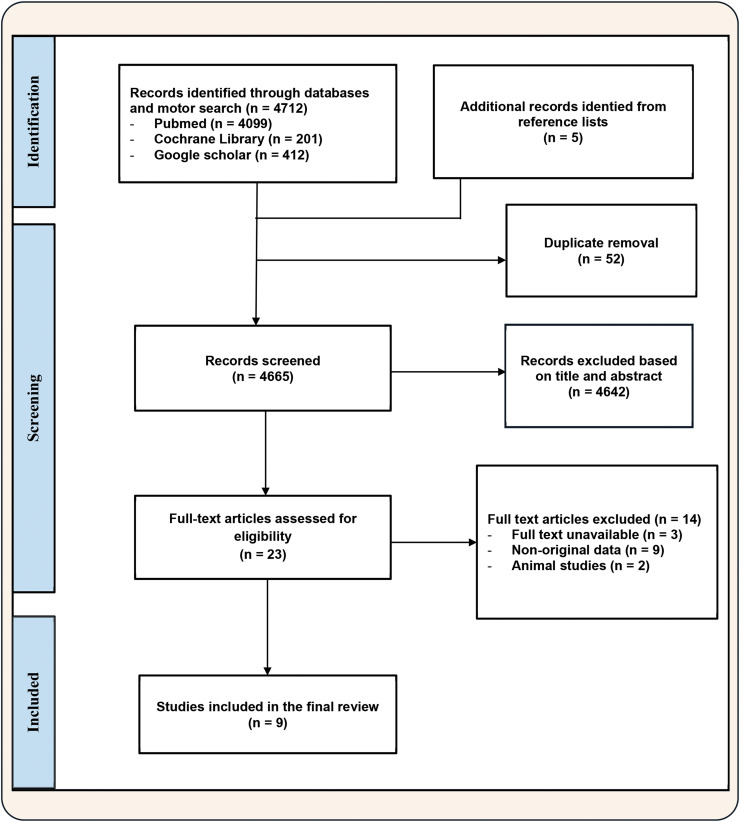
PRISMA flowchart for identifying published studies that were included in this review.

In the end, nine studies fulfilled all eligibility criteria and were included in the systematic review. These were illustrated in the PRISMA flow diagram (Fig. 1) and detailed in Table 1.

**Table 1:** Summary of reported studies on the surgical reconstruction of cerebral lymphatic drainage pathways. Including an identification of targeted pathologies and explored surgical approaches.

Authors Date	Study design	Title	Targeted pathologies	Explored surgical approach
Xie Q et al., 2023 [11]	Case report	Rewiring the Brain: The Next Frontier in Supermicrosurgery	Alzheimer’s disease	Lymphatico-venous anastomoses
Li X et al., 2024 [12]	Case report	Promising Outcomes Five Weeks After a Surgical Cervical Shunting Procedure to Unclog Cerebral Lymphatic Systems in an Alzheimer’s Disease Patient	Alzheimer’s disease	Lymphatico-venous anastomosis
Chen L et al., 2025 [13]	Case report	Perioperative care and recovery outcome of deep jugular venous lymphatic anastomosis in Alzheimer’s disease: A case report	Alzheimer’s disease	Lymphatico-venous anastomosis
Chen JY et al., 2025 [14]	Cohort study (single arm)	Deep cervical Lymphovenous anastomosis (LVA) for Alzheimer’s disease microsurgical procedure in a prospective cohort study	Alzheimer’s disease	Modified lymphatico-venous anastomosis technique
Xie Q et al., 2025 [15]	Case report	Potential Role of Lymphovenous Bypass in mitigating Alzheimer’s Disease Dementia	Alzheimer’s disease	Lymphatico-venous anastomosis
Hong JP et al., 2025 [16]	Narrative review	A Proposed Role for Lymphatic Supermicrosurgery in the Management of Alzheimer’s Disease: A Primer for Reconstructive Microsurgeons	Alzheimer ‘s disease	Lymphatico-venous anastomosis
Yang et al., 2025 [17]	Narrative review	Deep Cervical Lymphovenous Bypass for Parkinson’s Disease: A Hypothesis	Parkinson’s disease	Lymphatico-venous anastomosis
Ma Y et al., 2025 [18]	Narrative review	Deep cervical lymphaticovenous anastomosis in Alzheimer’s disease: A promising frontier or premature enthusiasm?	Alzheimer’s disease	Lymphatico-venous anastomosis
Wang H et al., 2025 [19]	Narrative review	Lymphatic-venous anastomosis surgery for Alzheimer’s disease	Alzheimer’s disease	Lymphatico-venous anastomosis

### 3.2 Clinical investigations

#### 3.2.1 Study and patient characteristics

A total of nine relevant publications published since 2023 were identified, including one single-arm cohort study, four case reports exploring the lympho-venous bypass surgery in the management of AD [[11], [12], [13], [14], [15]], and four narrative reviews on the subject [[16], [17], [18], [19]].

Across these studies, 30 patients were analyzed with a mean age of 70,5 years (range: 54–84), and a female predominance (20 women and 10 men, sex ratio 2:1). All patients were Chinese, diagnosed with advanced-stage AD, and underwent surgery. The average follow-up period was 1.4 months (range: 1–8 months), as reported in five of the studies.

#### 3.2.2 Surgical techniques

Only three studies described their surgical procedure aimed at replicating a lympho-venous shunt at the level of the deep cervical lymphatic vessels (DCLVs).

These approaches involved a cervical incision along the posterior border of the sternocleidomastoid (SCM) muscle to expose the lymphatic structures adjacent to the jugulocarotid axis. Under a surgical microscope, the DCLVs and deep cervical lymph nodes (DCLNs) were identified using near-infrared fluorescence imaging following indocyanine green (ICG) injection along the carotid sheath near the jugular foramen, as reported by Chen JY et al. et Xie Q et al. [14,15]. However, the team led by Chen L et al. did not specify the location of the cervical approach or the site of ICG injection [13].

Regarding the LVA techniques:

- Xie Q et al. used the “Octopus technique”, which enables the anastomosis of multiple small-caliber lymphatic vessels to a single vein. They also performed complementary lymph-node-to-vein anastomoses (LNVA) using branches of the external jugular vein (EJV).
- Chen JY et al. described a simplified modified LVA procedure in 26 patients, connecting a “lymphatic flap” compose on DCLVs and DCLNs from cervical drainage zones II, III, V to either the EJV or, alternatively, the internal jugular vein (IJV).
- Chen L et al. performed end-to-end anastomoses between the proximal portion of the DCLVs and the distal segment of an adjacent vein.

In all approaches, the patency and functionality of the anastomosis were confirmed by the passage of ICG into the venous system.

#### 3.2.3 Outcomes

The data collected reported across studies demonstrated marked heterogeneity in the clinical cognitive, imaging, and biomarker parameters assessed, as well as in the methods and follow-up durations. Despite this, a consistent trend toward early postoperative improvement was observed, typically within the first month after surgery. Cognitive benefits were reflected by modest increases in MMSE scores, ranging from +2 to +5 points, and in some cases MoCA scores (up to +2 points). One study reported in a video-documented improvements in memory, language, and registration subdomains [11]. Chen JY et al. observed at one month a statistically significant MMSE increase of +2 points (*p* = 0022), a non-significant MoCA improvement for 15 % patients, and a reduction in NPI scores in 42 % of patients. CSF Biomarkers analysis in a subset of 18 patients showed a trend toward decreased levels at 5–7 days postoperatively [14]. Functional improvements were also noted in ADCS-iADL and depression scores [12,13]. Imaging studies, including 18F-FDG and 18F-AV-45 PET scans, revealed increased glucose metabolism and reductions in cerebral tau and B-amyloid burden [12,15]. These findings suggest a potential short-term benefit of LVA surgery in selected patients with advanced AD.

#### 3.2.4 Complications

Only one study reported postoperative complications beyond episodes of transient confusion observed in the immediate postoperative period. In their cohort of 26 patients, Chen JY et al. described two cases of upper extremity paresis characterized by impaired arm mobility [14]. Symptoms gradually improved during follow-up and were likely related to accessory nerve injury sustained during surgical dissection.

#### 3.2.5 Quality assessment

The quality of the case reports was assessed using the Joanna Briggs Institute (JBI) Critical Appraisal Checklist for Case Reports. Across the studies, we observed substantial heterogeneity in both methodological rigor and outcome reporting, which limited comparability and overall interpretability. In addition, we assessed the methodological quality of the single-arm-cohort-study using the MINORS tool. This study received a score of 10 out of 16, indicating intermediate methodological quality [14]. Several sources of biases were identified and discussed by the authors. The study was non-comparative, with a limited sample size, and a short follow-up period of only one month. Furthermore, outcome assessment was not blinded.

### 3.3 Overview of the litterature

Our search identified four relevant narrative reviews investigating the potential of lymphatic surgery in the treatment of AD and PD.

In their 2025 review, Hong et al., explore the potential of lymphatic supermicrosurgery for AD, based on recent insights into cerebral lymphatic drainage [16]. They propose that extracranial lymphatic reconstruction could improve the clearance of Aβ and tau, potentially slowing disease progression. The authors highlight studies reporting cognitive improvement in lymphedema patients following LVA, and present emerging techniques such as lymphatic regeneration using nanofibrillar scaffolds. They also suggest that side-to-end anastomoses may be offer superior lymphatic flow restoration. However, they underline current limitations including unclear physiological mechanisms and potential complications like fibrosis or thrombosis, which require further investigations.

Similarly, Ma et al. emphasize the potential of LVA to restore the brain’s metabolic clearance pathways [18]. Their review highlights the limited nature of existing data, the high number of ongoing trials, and several preliminary observations, suggesting improvements in intracranial metabolic waste clearance, although the evidence remains anecdotal.

Wang, Levey, and Wang (2025) describe the implementation of LVA for AD across more than 30 clinical centers in China, with over 500 procedures performed since late 2024 [19]. While early clinical observation suggests possible cognitive benefits, the authors stress the lack of robust data regarding long-term efficacy or sustainability. Given the multifactorial nature of AD, they argue that LVA should be integrated with complementary approaches, such as anti-amyloid therapies, cognitive rehabilitation, and lifestyle modifications, to achieve synergistic benefits.

Finally, Yang et al. (2025) propose the deep cervical lympho-venous bypass (DCLB) as a novel surgical intervention for PD, aiming to facilitate the removal of α-synuclein aggregates by enhancing lymphatic clearance [17]. The procedure involves anastomosing DCLVs to cervical veins, under fluorescent imaging guidance, with bypasses ideally place on efferent vessels to preserve lymph node function. The authors also outline an experimental protocol in PD mouse models (A53T, 6-OHDA), including behavioral and histological analyses, MRI, inflammatory and metabolic markers.

## 4 Discussion

### 4.1 Cerebral lymphatic drainage

#### 4.1.1 Traditional pathway of CSF and cerebral waste clearance

**Fig. 2:**
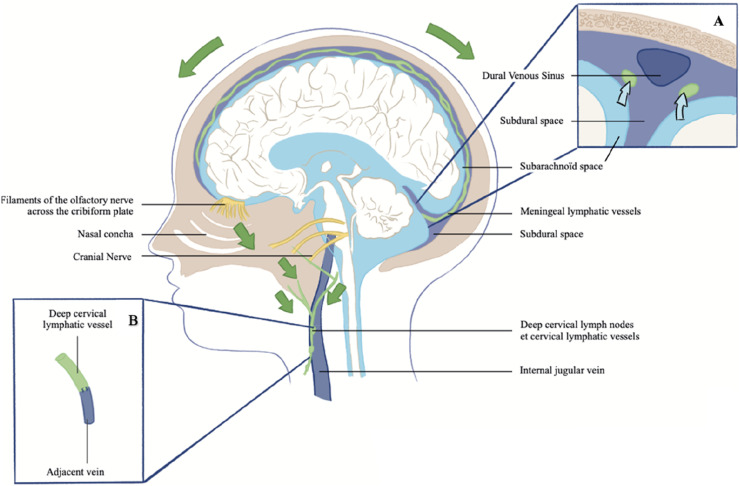
Schematic representation of the main cerebral lymphatic drainage pathways in humans. Green arrows indicate the direction of lymphatic flow toward the deep cervical lymphatic vessels and deep cervical lymph nodes. Inset A illustrates by the blue arrows the movement of a mixture of cerebrospinal fluid (CSF), interstitial fluid, and brain-derived waste products by the glymphatic or IPAD system from the subarachnoid space into the meningeal lymphatic vessels located in the subdural space adjacent to a dural sinus wall. Inset B shows a representation of a deep cervical lymphatico-venous anastomosis.

Initially, the traditional pathway of cerebral waste clearance was thought to mainly depend on CSF absorption through arachnoid granulations into venous sinuses [[20], [21], [22]]. However, recent evidence suggests the existence of additional clearance pathways, highlighting a more complex and diverse drainage network (Fig. 2) [[23], [24], [25]].

#### 4.1.2 The glymphatic system and its role in cerebral solute clearance

Described in 2012 by Iliff et al., the glymphatic system facilitates CSF circulation within the brain parenchyma. This pathway is based on communication between the subarachnoid and perivascular spaces, through which CSF flows convectively [1]. CSF follows the branching of the vascular network, penetrating through peri‑arterial spaces of penetrating arteries, propelled by arterial wall pulsatility [1,[26], [27], [28]]. It then crosses the glial basement membrane and astrocytic end-feet that express aquaporin-4 (AQP4), where it mixes with interstitial fluid (ISF) [1,28]. The resulting CSF/ISF mixture is drained toward perivenous and perineural spaces before reaching lymphatic vessels that border cranial nerves and meningeal lymphatics, carrying along cerebral metabolite [2,3,7,29,30]. This process is primarily regulated by AQP4, which is highly expressed in astrocytic end-feet adjacent to perivascular space [1,[31], [32], [33]].

Disruption of the glymphatic system has been linked to the abnormal buildup of metabolites (Aβ, phosphorylated tau, α-synuclein), which are associated with neurodegenerative diseases such as Alzheimer’s and Parkinson’s diseases [1,28,31,32,34,35].

#### 4.1.3 Controversy surrounding the glymphatic system

Despite considerable interest, the glymphatic model has encountered criticism. A study by Albargothy, Carare, et al. (2018) indicates that solute transport occurs through vascular basement membranes rather than perivascular space [36]. Furthermore, Smith and Verkman (2017) found that solute transport in the brain parenchyma is diffusive and independent of AQP4, thus questioning a fundamental aspect of the glymphatic system [37].

#### 4.1.4 The IPAD system as an alternative to glymphatic model

Weller and Nicoll (2004) originally proposed the concept of intramural peri‑arterial drainage (IPAD), describing it as the brain’s waste elimination system [38]. Carare et al. (2008) further supported this hypothesis, demonstrating that cerebral waste, particularly β-amyloid peptides, is eliminated via capillary basement membranes and arterial tunica media. Thus, IPAD is defined as a cerebral lymphatic mechanism [39].

Experimental microscopy studies have revealed a retrograde solute flow within arterial walls. Tracers injected into the brain parenchyma pass through capillary basement membranes, migrate along arterial membranes, and drain toward deep cervical lymph nodes or arachnoid villi [40].

Unlike the glymphatic model, IPAD depends on arterial pulsatility, though this has been questioned because of its excessive amplitude, which may not create the necessary pressure gradient [[41], [42], [43]]. Other mechanisms have been suggested, such as vasomotion, in which rhythmic contractions of vascular smooth muscle cells facilitate fluid movement within basement membrane [44], or the valve effect, where conformational changes in basement membranes may inhibit reflux and encourage unidirectional drainage [40].

Albargothy, Carare, et al. (2018) further emphasized how CSF and solutes infiltrate brain tissue through pia-glial basement membranes before being cleared via capillary and arterial basement membranes through the IPAD pathway. Their findings indicate that in aged mice, drainage is compromised, resulting in tracer accumulation in astrocytes and macrophages. This age-related impairment has been associated with cerebral amyloid angiopathy (CAA), in which Aβ peptides accumulate in vascular walls, further disrupting IPAD and contributing to neurodegenerative processes [36].

#### 4.1.5 Periolfactive and perineural lymphatic pathways

Perineural lymphatic drainage occurs via perineural lymphatic vessels that extend beyond the subarachnoid space after cranial nerves exit their foramina. These extracranial lymphatic vessels absorb CSF/ISF fluids from the subarachnoid space, which are then drained along neural pathways.

The peri‑olfactory lymphatic pathway, well-documented in various mammals and in humans, represents a significant drainage axis among these routes. These lymphatic vessels are located in the nasal submucosa, extending from the subarachnoid spaces along olfactory filaments after passing through the cribriform plate, capturing fluid outflow. They then connect to the peripheral lymphatic system through a nasopharyngeal lymphatic network, ultimately draining into dcLNs [7,21,[45], [46], [47], [48], [49], [50], [51], [52], [53], [54]]. This drainage route is not limited to fluid and solute transport, it also plays an immunological role by facilitating the migration of T cells and antigen-presenting cells into nasal lymphatic vessels and cervical lymph nodes, thus enhancing CNS immune surveillance [55]. However, recent MRI studies indicate that this drainage pathway is less pronounced in humans than in rodents [25].

Additionally, lymphatic vessels have been shown around other cranial nerves (CN II, CN V, CN VII, CN IX, CN X, CN XI) and spinal roots, aiding in CSF clearance and CNS immune regulation [7,24,46,56].

#### 4.1.6 Meningeal lymphatic vessels

The existence of meningeal lymphatic vessels (MLVs) has long been a topic of debate. However, in 2015, two independent teams rediscovered them, identifying these vessels in murine meninges using specific markers for lymphatic endothelial cells (LYVE-1, VEGFR2, PDPN, PROX1) [2,3,57]. These studies showed that MLVs play a crucial role in clearing the fluid mixture produced by the glymphatic system, as well as in transporting immune cells and solutes to the dcLNs.

In murine models, MLVs have been identified in the calvaria and at the base of the skull [3,6,58]. MRI confirmed their presence at these locations in humans, primarily within the dura mater, especially along the dural venous sinuses [48,59,60].

Unlike peripheral lymphatic vessels, MLVs form a less branched network of thin-walled initial lymphatic vessels that lack smooth muscle cells, suggesting distinct functional property [2]. Their valvular characteristics vary by location. Valves have been observed in basal MLVs, while those in the calvaria appear to be absent. This indicates that MLVs experience varying flow conditions based on their location, supporting the hypothesis of unidirectional flow toward the peripheral lymphatic system [2,3,7,58].

Although the exchange mechanisms between perivascular spaces, subarachnoid spaces, and MLVs remain poorly understood, several hypotheses have been proposed [61]. Ahn et al. (2019) suggest that fluid and macromolecule recapture occurs at “hot spots” in basal MLVs due to their proximity to the subarachnoid space and thinner dural layer [6]. In contrast, Ringstad and Eide (2020) propose that the parasagittal dura mater space could be an intermediate link between perivascular spaces and MLVs based on MRI observations showing CSF and solute drainage in this region [25].

### 4.2 Implications of the lymphatic system in cerebral waste clearance

#### 4.2.1 Impact of impaired cerebral lymphatic drainage on clearance mechanisms

Numerous experimental methods in murine models have shown that altering various lymphatic drainage pathways; whether through genetic blockade, chemical disruption, or surgical ligation of cervical lymphatic vessels; results in impaired clearance of cerebral waste, such as Aβ, phosphorylated tau, or α-synuclein [[2], [3], [4], [5],^35^,[62], [63], [64]]. Some of these studies have also indicated that blocking these pathways leads to cognitive deficits in animal models, suggesting a direct connection between lymphatic drainage and brain function.

In humans, a recent retrospective study by Chao et al. (2024), which involved 234 patients who underwent simple or bilateral cervical lymph node dissection, reported an increased risk of dementia, with a greater risk noted after bilateral dissection [65]. Although this study has limitations, its findings align with observations from experimental models.

Additional mechanism may contribute to impaired waste clearance, such as disrupted sleep architecture. Poor sleep quality is a well-established risk factor for cognitive decline in humans. Notably, activation of the glymphatic system occurs predominantly during the deep slow-wave sleep. In humans, a single night of sleep deprivation leads to increased Aβ levels, in the hippocampal, and thalamic regions, potentially reflecting reduced glymphatic function [66,67].

#### 4.2.2 Aging and impaired cerebral lymphatic drainage

Aging, the primary risk factor for neurodegenerative diseases such as Alzheimer's disease and idiopathic Parkinson's disease, has been directly implicated in cerebral lymphatic drainage dysfunction.

Observations in animal models and humans have identified a progressive alteration in glymphatic system function with age, including disrupted polarization of glial cells AQP4 adjacent to perivascular spaces and reduced arterial pulsatility, compromising glymphatic clearance efficiency [32,68]. These changes are accompanied by structural remodeling of MLVs, encompassing valve alterations, impairment of lymphatic endothelial cell junctions, reduced vessel caliber, decreased cerebral lymphatic coverage, and impaired waste clearance [4,6,7]. Additionally, degeneration of the nasopharyngeal lymphatic pathway, atrophy of deep cervical lymph nodes, and dysfunction of cervical lymphatic vessels have been documented [8,46,53]. In aged mice, MLVs display a hyperplastic phenotype, possibly indicating a compensatory mechanism responding to lymphatic hypertension similar to what is seen in peripheral lymphedema [6,60,69].

As a result, age-related degeneration of cerebral lymphatic pathways may gradually decrease drainage efficiency, hinder waste clearance, and lead to the accumulation of neurotoxic substances linked to neurodegenerative conditions.

### 4.3 Deep cervical lympho-venous bypass procedure

Since cerebral lymphatic drainage was identified, new therapeutic approaches, whether pharmacological, non-pharmacological, or surgical, have emerged, opening promising perspectives in managing numerous neurological pathologies.

Our current understanding of cerebral lymphatic dysfunction leads us to redefine it as a form of “cerebral lymphedema.” From this perspective, a surgical solution seems well-suited to restoring drainage pathways. Whether drainage predominantly occurs through the meningeal lymphatic system or via perineural lymphatic vessels, the flow converges toward the deep cervical lymphatic vessels and nodes. This convergence makes the cervical region the most appropriate target for surgically restoring cerebral lymphatic drainage.

Our systematic review highlights the emergence of a novel and promising therapeutic approach, with nine studies published in the past three years exploring deep cervical lymphatic surgery for neurodegenerative diseases. The work of Xie et al. (2023) marked the first clinical exploration on lymphatico-venous anastomoses applied to cerebral lymphatic drainage, paving the way for further investigation of this innovative procedure.

In July 2025, a total of 30 cases of deep cervical LVAs have been reported in the literature. Most studies describe moderate cognitive improvements observed as early as one month postoperatively. However, these results must be interpreted with caution due to substantial methodological limitations, high risk of bias, absence of control groups, short follow-up periods, and interventions performed at advances stages of Alzheimer’s disease. Collectively, these factors contribute to a low overall level of evidence. These limitations underscore the urgent need for higher quality data and support the development of prospective, comparative, and potentially randomized trials to robustly assess the clinical efficacy and long-term sustainability of LVA in this context.

Due to the limited number of published studies on this procedure, we expanded our search to include ongoing trials by consulting the ClinicalTrials.gov and Chinese Clinical Trial Registry (ChiCTR) databases. As of July 1, 2025, this search identified 25 additional clinical trials and two observational studies. These studies, conducted in Asia (China and Singapore), explore the efficacy and safety of deep cervical lymphatic shunts in the management of Alzheimer’s disease, and in one case, Parkinson’s disease (Table 2).

**Table 2:** Summary of ongoing or completed but unpublished, registered clinical trials investigating deep cervical lymphatico-venous anastomosis (LVA) for the Treatment of Alzheimer's Disease.

Public title	Study registration and Type	Location	Registration number	Surgical Technique	Patients (Surgery group / Control group)	Main Outcome	Study completion
Exploratory research on the improvement of brain function in Alzheimer's disease by cervical lymphatic vessel/node-vein anastomosis	2025–07–01 Interventional study	Shanghai Ninth People's Hospital, Shanghai JiaoTong University School of Medicine (China)	Chinese clinical trial registry ChiCTR2500105306	Deep cervical LVA and LNVA	*N* = 10 (Single group assignment)	Cognitive function and Quality of life	2027–04–30
Deep Cervical Lymphatic-Venous Anastomosis (LVA) for the Treatment of Alzheimer's Disease	2025–06–18 Interventional study	Baotou Central Hospital (China)	Chinese clinical trial registry ChiCTR2500104509	Deep cervical LVA and LNVA	*N* = 10 (Single group assignment)	Cognitive function and Quality of life	2030–04–26
Lymphatic-venous anastomosis for the treatment of Alzheimer's disease	2025–06–11 Observational study	Peking University Shenzhen Hospital (China)	Chinese clinical trial registry ChiCTR2500104139	LVA	*N* = 80 (20/60)	Cognitive function	2026–06–04
The Impact of Cervical Deep Lymphatic Venous Anastomosis on Biological Markers and Evaluation of Efficacy and Safety in Alzheimer's Disease	2025–05–19 Interventional study	The Second People’s Hospital of Guiyang (China)	Chinese clinical trial registry ChiCTR2500102675	Deep cervical LVA	*N* = 70 (35/35)	Cognitive function and Neuroimaging examination	2028–03–31
A Multicenter Randomized Controlled Study on the Efficacy of Deep Cervical Lymphovenous Anastomosis Combined with Pharmacological Treatment Versus Pharmacological Treatment Alone in Improving Cognitive Function in Patients with Alzheimer’s Disease	2025–05–19 Interventional study	Affiliated Hospital of Guangdong (China)	Chinese clinical trial registry ChiCTR2500102667	Deep cervical LVA	*N* = 92 (46/46)	Cognitive function and Quality of life	2028–11–30
Deep Cervical Lymphaticovenous Anastomosis Surgery for Moderate-to-Advanced Dementia Patients	2025–05–11 Interventional study	Renji Hospital, Shanghai Jiao Tong University School of Medicine (China)	ClinicalTrials.gov ID NCT06978946	Deep cervical LVA	*N* = 85 (NR/NR)	Cognitive function	2027–02–28
Clinical study on the safety, feasibility, and effectiveness of deep neck lymphatic vein anastomosis surgery for the treatment of moderate to severe Alzheimer's disease	2025–04–29 Interventional study	Changzhou No.2 People’s Hospital (China)	Chinese clinical trial registry ChiCTR2500101778	Deep cervical LVA	*N* = 40 (20/20)	Plasma AD biomarkers	2027–03–31
A Single-Center Prospective Clinical Study of Deep Cervical Lymphatic Vessel/Lymph Node-Vein Anastomosis for the Treatment of Alzheimer's Disease	2025–04–28 Interventional study	The First Affiliated Hospital of Xi'an Jiaotong University (China)	Chinese clinical trial registry ChiCTR2500101642	Deep cervical LVA / LNVA	*N* = 30 (Single group assignment)	Cognitive function and Quality of life	2027–02–28
An exploratory study of cervical deep lymphatic-venous anastomosis in the treatment of Alzheimer's disease	2025–04–27 Interventional study	Peking University School and Hospital of Stomatology (China)	Chinese clinical trial registry ChiCTR2500101614	Deep cervical LVA	*N* = 10 (Single group assignment)	Cognitive function and Quality of life	2027–04–30
Clinical Study of Lymphaticovenous Anastomosis (LVA) for the treatment of Alzheimer’s disease (AD)	2025–04–02 Interventional study	Peking University third hospital (China)	ClinicalTials.gov ID NCT06918145	Cervical LVA	*N* = 80 (Single group assignment)	Cognitive function	2030–03–01
Efficacy and Safety of Deep Cervical Lymphatic-Venous Anastomosis Combined with Lymphatic System Remodeling in the Treatment of Moderate and Severe Alzheimer's Disease	2025–03–31 Interventional study	China-Japan Union Hospital of Jilin University Committee (China)	Chinese clinical trial registry ChiCTR2500099855	Deep cervical LVA	*N* = 30 (15/15)	Cognitive function, Blood biomarkers, Serious adverse events	2026–03–01
Clinical Study on Deep Cervical Lymphatic Trunk Decompression Combined with Mid-Cervical Deep Lymph Node-External Jugular Vein Anastomosis for Alzheimer's Disease Treatment	2025–03–22 Interventional study	General Hospital of Tianjin Medical University (China)	ClinicalTrials.gov ID NCT06936514	Deep cervical LVA / LNVA	*N* = 45 (NR/NR)	Cognitive function and Neuroimaging examination	2025–10–31
Exploratory Study on the Improvement of Brain Function in Alzheimer's Disease by Cervical Lymphatic/Venous Anastomosis	2025–03–11 Interventional study	The Second Hospital of Jilin University (China)	Chinese clinical trial registry ChiCTR2500098639	Deep cervical LVA+LNVA	*N* = 10 (Single group assignment)	Cognitive function	2027–10–01
Deep Cervical Lymphaticovenous Anastomosis for Improving Neurological Function in Alzheimer's Disease Patients: A Single-Center, Prospective self-controlled trial	2025–03–06 Interventional study	The First Medical Center of Chinese PLA General Hospital (China)	Chinese clinical trial registry ChiCTR2500098356	Deep cervical LVA	*N* = 50 (Single group assignment)	Neuroimaging examination	2025–09–30
A Prospective, Multicenter Cohort Study of Cervical Deep Lymphovenous Anastomosis for the Treatment of Alzheimer's Disease	2025–02–21 Interventional study	Kunming Sanbo Brain Hospital (China)	Chinese clinical trial registry ChiCTR2500097585	Deep cervical LVA	*N* = 60 (30/30)	Cognitive function	2028–02–29
An Exploratory Study to Confirm Efficacy of Modified Deep Cervical Lymphovenous Anastomosis (LVA) in Alzheimer's Disease/​ Parkinson's Disease (SOLVEN)	2025–02–18 Interventional study	Zhejiang Provincial People's Hospital (China)	ClinicalTrials.gov ID NCT06852352	Deep cervical LVA	*N* = 160 (Single group assignment)	Cognitive function	2028–01–31
Clinical Application of Deep Lymphatic Drainage Therapy for Alzheimer's Disease	2025–02–14 Interventional study	Shandong Second Medical University First Affiliated Hospital (China)	Chinese clinical trial registry ChiCTR2500097251	Deep cervical LVA	*N* = 30 (Single group assignment)	Cognitive function, Neuroimaging examination, Blood biomarker	2027–03–01
Cervical Lymphatico-Venous Bypass for Treatment of Alzheimer's Disease - Proof of Concept Study (CLyVeB-AD-1 Study)	2025–01–17 Interventional study	Changi General Hospital (Singapore)	ClinicalTrials.gov ID NCT06965062	Deep cervical LVA or LNVA	*N* = 10 (Single group assignment)	Cognitive function and Quality of life	2030–03–31
Multicenter, Prospective Clinical Study on Deep Cervical Lymphatic-Venous Anastomosis for the Treatment of Moderate to Severe Alzheimer's Disease	2025–01–06 Interventional study	Harbin Medical University Affiliated Second Hospital (China)	Chinese clinical trial registry ChiCTR2500095309	Deep cervical LVA	*N* = 100 (Single group assignment)	Cognitive function and Quality of life	2027–12–31
Randomized controlled clinical trial of deep cervical lymphangiovenous anastomosis in the treatment of Alzheimer's disease	2024–12–25 Interventional study	The First People's Hospital of Zunyi City (China)	Chinese clinical trial registry ChiCTR2400094603	Deep cervical LVA	*N* = 100 (50/50)	Cognitive function and Quality of life	2027–10–31
Evaluation of comprehensive diagnosis and treatment efficacy of Alzheimer's disease based on bilateral cervical deep lymphatic vein anastomosis	2024–11–26 Interventional study	Zhengzhou Central Hospital (China)	Chinese clinical trial registry ChiCTR2400092975	Deep cervical LVA	*N* = 40 (20/20)	Cognitive function	2026–03–31
Efficacy and safety study of cervical lymphatic-vein bypass surgery in the treatment of Alzheimer's disease	2024–11–08 Interventional study	Dongguan Chashan Hospital (China)	Chinese clinical trial registry ChiCTR2400092047	Cervical LVA	*N* = 20 (10/10)	Neuroimaging examination	2025–09–11
A Single-Center, Prospective, Single-Arm Exploratory Study on Deep Cervical Lymphaticovenous Anastomosis for Improving Neurological Function in Alzheimer's Disease Patients	2024–09–19 Interventional study	Department of Neurosurgery, First Affiliated Hospital of Army Medical University (China)	Chinese clinical trial registry ChiCTR2400089883	Deep cervical LVA	*N* = 30 (Single group assignment)	Cognitive function / Quality of life	2027–08–10
Deep Cervical Lymphatlc-Venous Anastomosis Surgery for the Treatment of Alzheimer's Disease: A Pilot Study (DIVA Study)	2024–07–28 Interventional study	Zhejiang Provincial People's Hospital (China)	ClinicalTrials.gov ID NCT06530732	Deep cervical LVA	*N* = 60 (NR/NR)	Cognitive function	2026–09–30
Randomized controlled clinical trial of deep cervical lymphatic vessel vein anastomosis and lymphatic stemnectomy for the treatment of Alzheimer's disease	2024–05–21 Interventional study	The Ninth People's Hospital Affiliated to Shanghai Jiao Tong University School of Medicine (China)	Chinese clinical trial registry ChiCTR2400084617	Deep cervical LVA	*N* = 10 (5/5)	Cognitive function, Neuroimaging, LCS biomarkers	2025–11–30
An Exploratory Study of Deep Cervical Lymphovenous Bypass (LVB) in Alzheimer's Disease	2024–06–03 Interventional study	Affiliated Hospital of Jiangnan University (China)	ClinicalTrials.gov ID NCT06448975	Deep cervical LVA	*N* = 30 (Single group assignment)	Neuroimaging examination and Blood biomarkers	2026–07–01
A Pilot Study of Deep Cervical Lymphatic-venous Anastomosis in the Treatment of Alzheimer's Disease	2024–06–03 Observational study	Second Affiliated Hospital, School of Medicine, Zhejiang University (China)	ClinicalTrials.gov ID NCT06448442	Deep cervical LVA	*N* = 8 (Single group assignment)	Cognitive function	2028–08–01

In total, approximately 1300 patients, after removal of duplicate cohorts, are included or planned to be included in these studies. The evaluated outcomes are diverse, including clinical assessments of cognitive function, quality of life, neuroimaging examinations, measurement of blood and cerebrospinal fluid biomarkers, as well as safety and tolerability of the procedure.

Although the results have not yet been published, the large number of ongoing trials reflects the growing interest and enthusiasm of the medical community for this innovative approach.

#### 4.3.1 Lymphatico-venous anastomosis

**Fig. 3:**
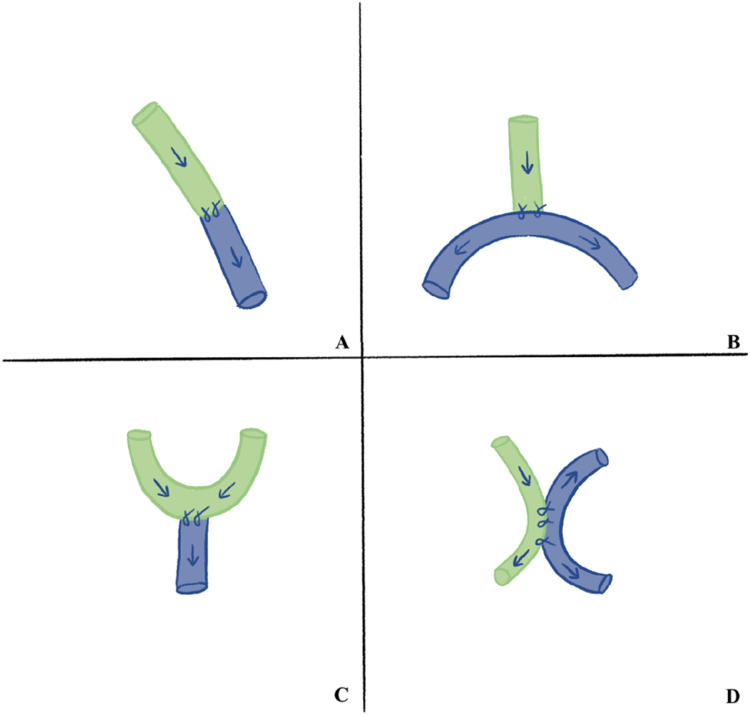
Schematic representation of the four main configurations of lymphatico-venous anastomoses (LVAs). (A) End-to-end; (B) End-to-side; (C) Side-to-end; (D) Side-to-side.

Supermicrosurgery represents a significant advancement in microvascular reconstruction, allowing for the anastomosis of vessels with diameters less than 0.8 mm, thereby expanding the technical limits of reconstructive surgery. Lymphatico-venous anastomosis, first described by Koshima et al. in the late 1990s, is now an established surgical option for treating peripheral lymphedema [70]. The principle of this technique is based on redirecting lymphatic flow from functional lymphatic vessels to an adjacent vein or venule of similar caliber, utilizing a pressure differential where venous blood pressure is lower than lymphatic pressure [71]. Four general types of anastomosis techniques are utilized: end-to-end, side-to-side, side-to-end, and end-to-side (Fig. 3).

Although LVA has been employed for several years in managing cervicofacial lymphedema, its application to deep cervical lymphatic vessels represents a significant technical challenge [[72], [73], [74], [75], [76]].

#### 4.3.2 Surgical considerations

Further evidence about the efficacy and safety of lymphatic reconstruction techniques is needed before they can be widely adopted in clinical practice. The refinement of LVA technical methods is still a topic of debate, even in the context of peripheral lymphedema management. Several factors need clarification to enhance this surgical approach and optimize therapeutic outcomes.

The effectiveness of LVA applied to the deep cervical lymphatic network depends on a favorable pressure gradient between the intracranial lymphatic network and cervical venous circulation. Cervical venous pressure is estimated to be between 0 and 6 mmHg, but we still lack precise measurements of the pressure gradient within the cerebral lymphatic system [16]. Ma et al. (2017) indicate in mouse model that lymphatic flow is propelled by a favorable pressure gradient between the subarachnoid space, contained within an inextensible cranial vault, and the cervical lymphatic vessels [7]. However, it remains unproven that such a gradient exists in human brain lymphatic system [16].

The functionality and caliber of targeted lymphatic vessels influence drainage effectiveness. Theoretically, targeting larger-diameter lymphatic vessels could allow more efficient drainage restoration. However, elevated endolymphatic pressure within cervical lymphatic vessels could induce structural changes similar to peripheral lymphatics, such as dilation, loss of smooth muscle cells (SMCs) contractility, valve dysfunction, scarring, and sclerosis [[77], [78], [79]]. Smaller-diameter vessels (approximately 0.5 mm) maintain better functional integrity, making them better candidates for an effective anastomosis [80]. Indocyanine green lymphography has proven to be a valuable method for identifying functional lymphatic vessels to target [81,82]. In the context of deep cervical LVA surgery, intraoperative injection of ICG near the jugular foramen along the carotid sheath appears to allow precise localization of functional downstream deep cervical lymphatics [14,15].

The literature data raises the question of whether unilateral or bilateral anastomoses should be realized. Murine studies show that after tracer injection into one cerebral hemisphere, drainage mainly occurs into the ipsilateral deep cervical lymph nodes, with minimal passage to the contralateral node [6]. Extrapolating to humans, and considering the bilateral involvement of the cerebral parenchyma, we propose that bilateral procedure is preferable to optimize drainage efficiency. In line with this rationale, current published studies and ongoing clinical trials are now exploring bilateral procedures to enhance the likelihood of therapeutic success.

In peripheral lymphedema, the optimal number of anastomoses is still unknown. The team led by Yi et al. (2024) showed that, in a cohort of 121 lower limb lymphedema patients, the greatest clinical benefit occurred when performing 6 to 8 anastomoses [83]. However, other studies found no correlation between the number of anastomoses performed and clinical outcomes [84,85]. Actually no consensus has been reached regarding the optimal number of anastomoses required to ensure the effectiveness of LVA-based-treatment. Regarding the anastomosis technique, some authors suggest that, technically, side-to-end LVAs could be more effective than the end-to-end technique due to a favorable pressure gradient and preservation of downstream lymphatic vessel functionality [16,86]. Pak, Hong, and colleagues (2021) demonstrated greater effectiveness when LVA was combined with lymph node venous anastomosis (LNVA) [87]. Moreover, integrating deep cervical LVA surgery with other procedures, such as anti-amyloid therapies, cognitive rehabilitation, and lifestyle modifications, may enhance therapeutic efficacy [19].

In advanced stages of Alzheimer's or Parkinson's diseases, irreversible neuronal damage may limit the therapeutic potential of surgical reconstruction techniques [88,89]. However, restoring lymphatic outflow in early or moderate stages could help prevent the accumulation neurotoxic solute, such as soluble Aβ, and slow the formation of amyloid plaques, thereby slowing clinical disease progression. Although current clinical applications have primarily focused on patients with late-stage disease, we hypothesize that this strategy would be more effective if implemented earlier in the course of neurodegeneration.

Robot-assisted surgery could greatly improve deep lymphatic-venular anastomoses (LVA). Recent reviews emphasize the benefits of robotic systems (MUSA and SYMANI), including reduced physiological tremors, enhanced control of micro-movements, better access to deep structures, and greater precision in surgical procedures [90,91]. These technological advancements may increase the reproducibility and safety of deep cervical LVA procedures.

#### 4.3.3 Safety and complications

Lymphatico-venous anastomoses conducted for peripheral lymphedema have shown a high level of intraoperative safety [92,93]. However, specific complications associated with LVA have been identified.

Venous reflux is associated with reduced treatment effectiveness [94,95]. A backflow of blood into the central nervous system could have serious consequences for patient safety. This risk is heightened given the uncertain presence and functionality of lymphatic valves in human meningeal lymphatics [16]. Several surgical techniques have been developed to mitigate these risks, including external valvuloplasty, Y-vein plasty, and peripheral venous angle plasty [94,[96], [97], [98]]. These methods, initially created for managing peripheral lymphedema and validated through the retrograde milking test, help alleviate lymphedema by eliminating venous reflux in treated patient groups.

The infectious risk is a significant concern, as these anastomoses could facilitate retrograde dissemination of pathogens into the central nervous system. This risk could be exacerbated by thrombosis formation within the anastomoses.

Finally, these anastomoses could facilitate metastatic dissemination of head and neck tumors, as well as primary brain tumors. Although no data are currently available regarding this risk of dissemination, this oncological risk imposes increased vigilance in the selection of eligible patients.

Due to the emerging nature of lymphatic flow restoration through surgical intervention, additional potential complications must be considered. This necessitates validation of the procedure’s safety through rigorous preclinical studies.

## 5 Conclusion

The understanding of cerebral lymphatic drainage has significantly evolved over recent decades, challenging traditional paradigms centered on arachnoid villi as the primary cerebrospinal fluid drainage pathway. Identifying lymphatic circuits has revealed complex networks involving macromolecule clearance, fluid homeostasis, and immune surveillance within the brain. Dysfunctions of these drainage systems are now recognized as contributing factors in the progression of neurodegenerative diseases, particularly Alzheimer’s and Parkinson’s disease. In these conditions, the accumulation of pathogenic macromolecules leads to neuroinflammation, neurotoxicity, and neurodegeneration with progressive decline in cognitive functions. These advancements have paved the way for innovative therapeutic strategies to enhance cerebral metabolic waste clearance. By considering these dysfunctions as a form of “cerebral lymphedema”, lymphatic reconstruction techniques, such as lymphatico-venous anastomoses, emerge as promising therapeutic options. Nevertheless, despite encouraging preliminary results, validating these techniques requires rigorous preclinical evaluations, anatomical feasibility studies, and clinical trials to establish their efficacy and safety before widespread application in clinical practice can be considered. Should therapeutic benefits be confirmed in the management of neurodegenerative diseases, extending these supermicrosurgical reconstruction techniques could offer an innovative therapeutic solution for other cerebral pathologies associated with impaired brain lymphatic system.

## Funding

No funding was received for this review.

## CRediT authorship contribution statement

**Theodore Lahmar:** Writing – original draft, Methodology, Investigation, Formal analysis, Data curation, Conceptualization. **Francois Thuau:** Writing – review & editing. **Gaelle Pinard:** Writing – review & editing, Visualization, Validation. **Claire Boutoleau-Bretonniere:** Writing – review & editing, Validation, Supervision, Data curation. **Pierre Perrot:** Writing – review & editing, Visualization, Validation, Supervision. **Ugo Lancien:** Writing – review & editing, Visualization, Validation, Supervision, Methodology, Data curation.

## Declaration of competing interest

The authors declare that they have no known competing financial interests or personal relationships that could have appeared to influence the work reported in this paper.
